# A cluster-based trusted routing method using fire hawk optimizer (FHO) in wireless sensor networks (WSNs)

**DOI:** 10.1038/s41598-023-40273-8

**Published:** 2023-08-11

**Authors:** Mehdi Hosseinzadeh, Joon Yoo, Saqib Ali, Jan Lansky, Stanislava Mildeova, Mohammad Sadegh Yousefpoor, Omed Hassan Ahmed, Amir Masoud Rahmani, Lilia Tightiz

**Affiliations:** 1 Institute of Research and Development, Duy Tan University, Da Nang, Vietnam; 2 School of Medicine and Pharmacy, Duy Tan University, Da Nang, Vietnam; 3 School of Computing, Gachon University, 1342 Seongnamdaero, Seongnam, 13120 Korea; 4 Department of Information Systems, College of Economics and Political Science, Sultan Qaboos University, Al Khoudh, Muscat, Oman; 5 Department of Computer Science and Mathematics, Faculty of Economic Studies, University of Finance and Administration, Prague, Czech Republic; 6 Department of Computer Engineering, Dezful Branch, Islamic Azad University, Dezful, Iran; 7 Department of Information Technology, University of Human Development, Sulaymaniyah, Iraq; 8 Future Technology Research Center, National Yunlin University of Science and Technology, Yunlin, Taiwan

**Keywords:** Climate sciences, Environmental sciences

## Abstract

Today, wireless sensor networks (WSNs) are growing rapidly and provide a lot of comfort to human life. Due to the use of WSNs in various areas, like health care and battlefield, security is an important concern in the data transfer procedure to prevent data manipulation. Trust management is an affective scheme to solve these problems by building trust relationships between sensor nodes. In this paper, a cluster-based trusted routing technique using fire hawk optimizer called CTRF is presented to improve network security by considering the limited energy of nodes in WSNs. It includes a weighted trust mechanism (WTM) designed based on interactive behavior between sensor nodes. The main feature of this trust mechanism is to consider the exponential coefficients for the trust parameters, namely weighted reception rate, weighted redundancy rate, and energy state so that the trust level of sensor nodes is exponentially reduced or increased based on their hostile or friendly behaviors. Moreover, the proposed approach creates a fire hawk optimizer-based clustering mechanism to select cluster heads from a candidate set, which includes sensor nodes whose remaining energy and trust levels are greater than the average remaining energy and the average trust level of all network nodes, respectively. In this clustering method, a new cost function is proposed based on four objectives, including cluster head location, cluster head energy, distance from the cluster head to the base station, and cluster size. Finally, CTRF decides on inter-cluster routing paths through a trusted routing algorithm and uses these routes to transmit data from cluster heads to the base station. In the route construction process, CTRF regards various parameters such as energy of the route, quality of the route, reliability of the route, and number of hops. CTRF runs on the network simulator version 2 (NS2), and its performance is compared with other secure routing approaches with regard to energy, throughput, packet loss rate, latency, detection ratio, and accuracy. This evaluation proves the superior and successful performance of CTRF compared to other methods.

## Introduction

Wireless sensor networks (WSNs) contain hundreds or thousands of sensors that are scattered over a large area. They control the environment and process the collected data in an efficient and distributed manner^1,2^. These networks have many applications, for example, military field (to detect and arrange forces in enemy land), detection of biological, chemical, and nuclear radiation, environmental monitoring and protection, agricultural monitoring, and industrial product control^6,7^. In addition, WSNs are used in smart homes, smart transportation, smart cities, and the protection of cultural and commercial monuments^8,9^. In WSNs, the nodes are equipped with a battery, which is hardly recharged or replaced. These nodes consume a lot of energy when sensing, processing, and sending environmental information. Compared to data processing and sensing, data transfer requires more energy^3,4^. For this reason, energy consumption management is essential in WSN. One of the effective solutions to achieve this goal is clustering. In a clustered topology, the energy efficiency is increased and the communication bandwidth is also preserved^5,8^. In this topology, the network is divided into different clusters and sensor nodes play different roles. In this case, the selection of cluster heads is a very important challenge. Therefore, it is very necessary to investigate new solutions such as meta-heuristic algorithms to address this challenge. Cluster-based routing methods define two communications, including intra-cluster communication and inter-cluster communication. These methods are scalable and efficient in terms of energy consumption and improve network lifetime. Nowadays, researchers have done a lot of research on cluster-based routing for WSNs. However, these research works still need to be further improved and adapted to the WSN environment because WSN, compared with traditional networks, have many restrictions such as limited resources, unreliable communications, operations without supervisor, and the lack of central management^10,11^.

Additionally, valid sensor nodes must perform the data transfer operation since data packets may be destroyed because of missing, interference, or sabotage by attackers^12,13^. For this reason, security must be addressed to protect the transmitted data packets against various attacks. Today, trust-based solutions are useful to deal with malicious nodes in WSN. Trust is especially important in cyber security. It evaluates the security status of sensor nodes based on their behaviors and interactions and actively separates normal nodes from hostile nodes, and thus it thwarts the security risks of these hostile nodes, such as privacy violations, changes in data, and conspiracy to organize more sophisticated attacks^14,15^. Therefore, the design of security mechanisms is a very important challenge in high-risk and insecure WSN environments and researchers cannot ignore security in the data transmission process. There is a contradiction between security and energy consumption. On the one hand, strong security methods are responsible for designing security techniques in sensor nodes to securely send data to the base station. On the other hand, sensor nodes are faced with limited energy resources and cannot run strong and complex security systems to ensure security in the data transmission process. When designing WSN protocols, it is necessary to combine security and energy efficiency to achieve an energy-efficient and lightweight trusted routing process. Achieving security and energy efficiency at the same time causes researchers to focus on cluster-based trusted routing methods, and they have done a lot of research to design a secure and appropriate routing method in wireless sensor networks. However, these methods still need to be improved.

In summary, this paper addresses existing research challenges and presents a new secure routing approach. The purpose of the method is to enhance network security by considering the limited energy of nodes in WSNs. To achieve this goal, a cluster-based trusted routing scheme using the fire hawk optimizer called CTRF has been introduced. In CTRF, a weighted trust mechanism (WTM) is designed in accordance with the interactive behavior of nodes. The main feature of WTM is to use a regulatory coefficient for trust parameters. This coefficient reduces or increases the trust level of sensor nodes according to their hostile or friendly behaviors. Moreover, CTRF presents a clustering mechanism based on the fire hawk optimizer (FHO). This mechanism is responsible for selecting cluster head nodes. In the clustering process, a new cost function is proposed to evaluate responses. Finally, CTRF creates inter-cluster paths through a trusted routing algorithm and uses these paths to transmit data from cluster heads to the base station. The main contributions of this paper are listed as follows:

- Presenting a weighed trust mechanism (WTM) to estimate the trust of nodes with regard to weighted reception rate, weighted redundancy rate, and energy state.
- Designing a clustering mechanism based on the fire hawk optimizer to select cluster head nodes.
- Introducing a trusted routing mechanism to determine inter-cluster paths.
- Comparing CTRF and other routing schemes based on energy, throughput, packet loss rate, latency, detection rate, and accuracy.

The structure of the paper is arranged as follows: In “Related works”, some secure routing methods are introduced in WSNs. “Basic concepts” briefly describes optimization algorithms, especially, fire hawk optimizer (FHO). “System settings” expresses the system settings, including the network model, the energy model, and the attack model. In “Proposed scheme”, the proposed routing scheme is introduced in wireless sensor networks. “Simulation and result evaluation” presents and analyzes the simulation results. Finally, the most important conclusions of the paper are stated in “Conclusion”.

## Related works

In Ref.^16^, a secure routing approach is proposed for wireless body sensor networks (WBSNs). This approach is called SecAODV, which includes three segments: bootstrap operation, inter-cluster path formation, and security. In the bootstrap operation, the base station has the responsibility to load the main instructions and encryption functions in the storage space of nodes. Moreover, in the inter-cluster path formation, cluster heads calculate their degree with regard to parameters like distance, remaining energy, connection quality, and hop counts to decide on broadcasting route request messages. In the security segment, a symmetric encryption strategy is responsible for protecting connection links within clusters. In addition, an asymmetric encryption strategy has the responsibility to protect connections between cluster heads. The simulation results show that SecAODV has improved delay, throughput, consumed energy, and packet delivery/loss rates.

In Ref.^17^, authors have introduced an energy-efficient secure routing scheme for the Internet of Things-based wireless sensor network. In this scheme, BS can detect attackers at the data verification phase. Additionally, the aggregator nodes have the responsibility to forward data from nodes to BS safely and securely. This method uses a secret data sharing technique, which has been improved by the bit-wise XOR-based encryption, hash functions, and network features. The authors have designed the method to protect the network against reply attacks, modification attacks, selective forwarding attacks, and data leakages. In addition, this scheme especially makes a tradeoff between network longevity and data security because IoT devices have constrained resources and low communication capabilities. The simulation process indicates that this scheme performs better than Sign-share, Sham-share, and PIP in terms of the required time and the consumed energy in the data processing operation.

In Ref.^18^, a dynamic trust system based on a recommendation filter strategy is suggested in the Internet of Things (IoT). This approach can increase the trust evaluation speed because it obtains direct trust based on a sliding window and a time decay function. Additionally, a recommendation filter technique has been used to effectively separate bad recommenders and reduce the negative effect of malicious devices. To merge direct trust and the recommended trust, an adaptive weighted coefficient has been considered. The experimental results indicates that this trust system rises the convergence speed and lowers mean error rate in comparison with TBSM, NRB, and NTM. Also, it has good resistance to attacks.

In Ref.^19^, a fuzzy logic-based and temperature-aware clustered routing approach has been introduced for WBANs. This method utilizes a fuzzy logic controller (FLC) to arrange sensors in clusters and then uses a data aggregation technique. The authors consider several scales, including CH temperature, the number of same neighbors, the number of neighboring nodes, residual energy, and the breakage of routes in the clustering process. In addition, this method proposes another FLC, which is responsible for coordinating patients when sending data from cluster heads to the coordinator. This FLC is designed with regard to factors, like distance, the number of patients linked to the coordinator, and PDR. The authors have designed a new optimization algorithm called HAOA to adjust factors and fuzzy rules in FLCs and enhance their performance. HAOA is inspired by the AOA algorithm, and its aim is to reduce local optimum problem and convergence rate. The simulation results prove the successful performance of this method and its ability to increase network stability and lifetime.

In Ref.^20^, a layered routing algorithm using the gray wolf optimization algorithm called LBR-GWO is proposed. The end of LBR-GWO is to rise network longevity. In this approach, the nodes are categorized into four layers. In layer one, the nodes are selected as cluster heads. Now, if there are more than two nodes in the first layer, cluster heads are chosen using a game theory-based system. Otherwise, the CH selection is done with regard to the remaining energy of the nodes. Compared to other methods, LBR-GWO is suitable for clustered networks. Simulation results prove that LBR-GWO balances energy consumed by nodes and improves network longevity compared to LEACH, HEED, and PSO.

In Ref.^21^, a three-level trust evaluation technique is introduced to accurately detect malicious nodes using a secure routing algorithm based on the grey wolf optimization (GWO). This scheme is named 3LWT-GWO. This approach consists of three steps: (1) Trusted clustering process (2) CH selection, and (3) Optimized routing operation. In the trusted clustering process, each node computes an overall trust score (OTS), which is a combination of several trust factors, including direct trust, indirect trust, energy, long-term recommendation, authentication, and connection quality. After finding insecure nodes, network nodes are grouped in clusters using a trusted clustering process. In this step, each node obtains a weight value with regard to its remaining energy, its distance to nodes, and the total energy. This weight value is used for selecting cluster heads. Finally, the optimized routing operation finds the optimal path based on GWO, which depends on trust degree, distance, latency, and distance. Then, data packets are forwarded to the desired node through the path. Given the simulation results, it can be said that 3LWT-GWO has a successful performance.

In Ref.^22^, an activation function-based trust-aware routing approach called AF-TNS is suggested in WSNs. This approach is implemented in two steps: energy-restricted trust evaluation and additive metric-based node evaluation to protect the trust of neighboring nodes. AF-TNS utilizes a random Transigmoid function to decide on secure and insecure nodes to maintain network stability. The simulation results indicate that AF-TNS improves detection rate and network lifetime.

In Ref.^23^, a secure atom search routing (SASR) technique, inspired by molecular dynamics behavior is proposed. SASR offers an effective solution to solve optimization problems. Additionally, SASR makes a tradeoff between exploitation and exploration and improves network performance. In SASR, there is a knowledge base, which is responsible for processing data, this reduces the computational complexity and increases network longevity. The simulation results show that SASR has improved network performance with regard to scales such as trust, delay, throughput, energy, PDR, and network longevity.

In Ref.^24^, a radial-shape clustering (RSC) algorithm is introduced in WSNs. RSC is a complex clustering structure, which divides the deployment environment into several virtual rings. Then, these rings are grouped into groups called clusters. RSC can solve energy and scalability issues. In RSC, cluster heads are chosen from sensor nodes close to the center of each cluster. In this method, intra-cluster communication is done using a one-hop routing. Also, a multi-hop or hierarchical routing technique is considered for inter-cluster communication so that the data aggregated by each CH will be transferred to the sink node through other cluster heads. Hence, CHs aggregate data obtained from cluster members and use an angular inclination routing technique to forward the aggregated data to the sink node. The results obtained from the simulation process display the successful performance of this method. Also, RSC has good performance in scalability and network lifetime.

In Ref.^25^, the authors seek to detect selfish nodes in a dynamic ad hoc network (DANET). Selfish nodes are malicious nodes, which damage the network performance because they do not participate in the routing process and conserve their resources, especially energy and memory. Selfish nodes threaten data accessibility and increase latency and routing overhead in the routing process. The proposed method in Ref.^25^ uses an evidence-based detection to identify the malicious nodes in the network. Also, trust authority (TA) has responsibility to detect the replicated nodes created by attackers. It builds a self-centered friendship (ISCF) tree. Then, the replica is determined for each the node based on the number of data accesses and the level of the node. This scheme can detect selfish nodes accurately and quickly and improves routing cost in the network.

In Ref.^26^, a trust-based routing protocol called CTEA is proposed for WSNs. It considers communication trust and is aware of energy consumed by sensor nodes in the network. CTEA counteracts two attacks, namely badmouth and energy drain attacks. These attacks threaten energy efficiency and negatively affect network lifetime. CTEA aims to improve network reliability, energy efficiency, and security by using the Dempster theory. In CTEA, the Dempster theory has been used to model the trustworthiness of nodes in the network based on their past behavior and to evaluate communication reliability. Moreover, CTEA considers energy metric to determine the routing paths between source and destination. The simulation results demonstrate that CTEA has acceptable performance and enhances network lifetime because it can timely detect energy drain and bad mouth attacks.

Table 1 presents strengths and weaknesses of the related works.

**Table 1:** Comparison of the related works.

Scheme	Strengths	Weaknesses
SecAODV^16^	Using a hierarchical topology, considering energy efficiency and network security at the same time, regarding the remaining energy of nodes when finding new paths, using a hybrid cryptography to secure connections between nodes	High communication cast, high delay in the routing process
Shivhare et al.^17^	Counteracting reply attacks, modification attacks, selective forwarding attacks, and data leakages, making a tradeoff between network longevity and data security, considering energy efficiency in the routing process	Low scalability, need to more experiments
Chen et al.^18^	Designing an adaptive trust model, using a recommendation filtering algorithm, high accuracy, high trust evaluation speed	High communication overhead, high time complexity
Javaheri et al.^19^	Using fuzzy logic in the clustering process, considering a hierarchical topology, adjusting factors and fuzzy rules based on HAOA, considering energy efficiency in the clustering and routing processes, increasing network stability and lifetime	Not designing a security mechanism for separating abnormal nodes from normal nodes, high time complexity
LBR-GWO^20^	Increasing network lifetime, considering energy efficiency in the clustering, considering a hierarchical topology, high scalability	Not designing a security mechanism for separating abnormal nodes from normal nodes, high time complexity, not considering inter-cluster routing
3LWT-GWO^21^	Designing a three-level trust evaluation technique, considering a hierarchical topology, high scalability, regarding energy efficiency in the clustering, high detection rate, high accuracy	Low convergence speed, need to improve data aggregation process
AF-TNS^22^	Selecting trusted neighbors in the secure transmission process, using a simple decision-making system, high network stability	Designing a weak trust system, low scalability, not considering a clustering process
SASR^23^	Selecting an optimal and secure route between source and destination, low computational complexity, considering a hierarchical topology, regarding energy efficiency in the clustering, improving network lifetime	High delay in the data transmission process, high communication cost
CRSC^24^	Balanced energy distribution between sensor nodes, increasing network lifetime, high scalability and reliability in the routing process	High time complexity, choosing cluster heads only based on distance and ignoring other parameters, especially energy in the CH selection process
Gopal and Saravanan^25^	Guaranteeing data accessibility, high accuracy, high detection speed, low delay and low communication cost in the routing process	Not considering energy in the routing process, not considering energy efficiency in the security mechanism, ignoring the clustering mechanism to increase scalability
CTEA^26^	Designing a strong trust mechanism, detecting badmouth and energy drain attacks, considering communication trust and improving security and reliability in the data transmission process, considering energy efficiency, improving network lifetime	High computational complexity, low scalability

## Basic concepts

Metaheuristic algorithms based on biological computing are advanced search algorithms, which are used to solve complex problems in different areas. These algorithms simulate the swarm intelligence (SI) concept in the social behavior of living things and are called SI-based systems. Today, these SI-based methods are widely used to solve problems such as clustering and routing in wireless sensor networks^27,28^. They have improved significantly the performance of these networks. In 2022, Azizi et al. proposed a SI-based algorithm called the fire hawk optimizer (FHO), which simulates the behavior of fire hawks when finding food and hunting prey. In FHO, fire hawks and preys play the role of candidate responses, and their position is refreshed at each step of the algorithm to get an optimal response (the main fire). In addition, FHO does not refresh the responses (fire hawks and prey) only based on the best response with the highest fitness (the main fire), but also it considers other responses with high fitness (fire hawks) in the position updating process. To determine the new territory of each fire hawk, the position of other hawks is considered to prevent the entrapment of the FHO algorithm in the local optimum. In this regard, responses will eventually converge to a global optimum. See Ref.^29^ for more details. In CTRF, the FHO algorithm is used to design an efficient clustering technique because finding the best cluster heads among sensor nodes in WSNs, especially large-scale networks, is known as an optimization problem. Hence, solving this problem using simple mathematical techniques is not easy and requires a high time. In the current research, FHO has been chosen to solve this optimization problem because various tests have proven its efficiency and competence compared to other meta-heuristic algorithms. In Ref.^29^, the authors have conducted several experiments and compared FHO with other meta-heuristic algorithms such as bat-inspired algorithm (BIA), butterfly optimization algorithm (BOA), whale optimization algorithm (WOA) algorithm, grey wolf optimizer (GWO), and the ray optimization algorithm (ROA). They have confirmed that FHO is a good and effective algorithm for solving various optimization problems. Note that FHO has a high ability in making a balance between exploration and exploitation and is capable in finding the global optimum. It has many advantages such as simplicity, free-parameter, high convergence speed, and global optimal search ability. Furthermore, it is beneficial to avoid falling into a local optimum. The points mentioned above are our main reasons for applying FHO in the clustering mechanism in CTRF.

## System settings

Here, the network model, the energy model, and the attack model are explained.

### Network model

**Figure 1:**
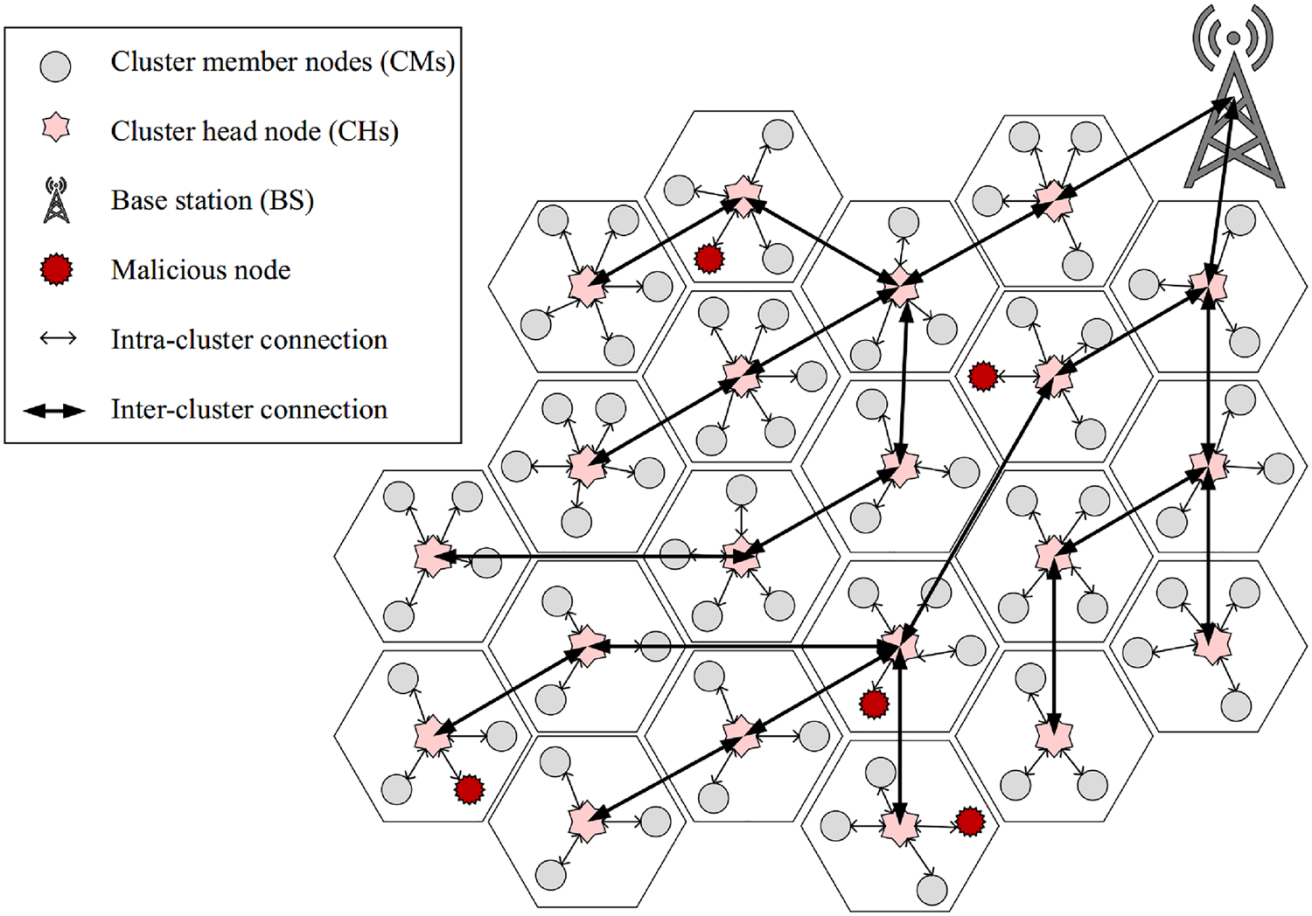
Network model in CTRF.

Figure 1 depicts the network model in CTRF. When launching the network, all nodes (i.e. $sn_{1},sn_{2}, \ldots ,sn_{i}, \ldots ,sn_{N}$) can be cluster head (CH) or cluster member (CM). In this network, CMs first sense the environment and deliver the collected data to their CH. Next, this CH performs the data aggregation operation and transfers the aggregated data to the base station (BS) through a certain route. Once the aggregated data is received by BS, it analyzes them and issues the relevant commands based on the network conditions. The assumptions of the network are summarized below:

- The base station is fixed, and its location is predetermined in the network.
- The base station has unlimited energy and high processing power.
- All nodes are informed of the BS location on the network.
- All nodes are homogeneous and are known by a specific ID (i.e. $ID_{sn_{i}}$).
- The nodes are motionless and are randomly scattered on the network.
- A positioning system is installed on all nodes to determine their position.

### Energy consumption model

In WSNs, a challenging issue is to optimize the consumed energy of nodes because these nodes are equipped with a tiny battery that is not easily replaced or recharged. Each node performs various operations such as sensing, processing, data storage, and receipt/sending. Communication operation is an energy-consuming operation in sensor nodes. According to this energy model, the consumed energy to exchange each $k-bit$ frame between $sn_{i}$ (receptor) and $sn_{j}$ (sender) is calculated as follows.

Note that the distance between $sn_{i}$ and $sn_{j}$ is *d*. The energy employed by $sn_{i}$ is obtained from Eq. (1):

1 $$\begin{aligned} {E_{TX}}\left( k,d \right) =\left\{ \begin{matrix} {E_{elec}}\times k+{E_{fs}}\times k+{d^{2}},\,\,\,\,\,\,\,d<{d_{0}} \\ {E_{elec}}\times k+{E_{mp}}\times k+{d^{4}},\,\,\,\,\,\,\,d\ge {d_{0}} \\ \end{matrix} \right. \end{aligned}$$

Moreover, the energy employed by $sn_{j}$ is expressed in Eq. (2):

2 $$\begin{aligned} {E_{RX}}\left( k,d \right) ={E_{elec}}\times k, \end{aligned}$$

where, ${E_{elec}}$, ${E_{fs}}$, and ${E_{mp}}$ indicate the energies employed in the electrical board, amplifier in the free space, and amplifier in the multi-path model, respectively. Also, ${d_{0}}$ is the distance threshold and is equal to ${d_{0}}=\sqrt{\frac{{E_{fs}}}{{E_{mp}}}}$.

### Attack model

Given the specific features of WSNs, for example, dynamic topology, deploying in adverse and out-of-reach areas, and the lack of a central controller, it is almost impossible to monitor these networks continuously^30,31^. Moreover, attackers may access, manipulate, or change the information exchanged between sensor nodes because the connection links between these nodes are wireless. Thus, these networks are highly exposed to cybersecurity attacks^32^. This proves the necessity of a secure routing protocol. CTRF focuses specifically on two black hole and flooding attacks. A black hole attacker is different from normal nodes, and the most important difference is that if a black hole node gets a route request (RREQ) from each node, it does not check whether it has a real route to the desired node, and quickly creates a route reply (RREP) message and returns it to the source node. This attacker adjusts the parameters related to this fake RREP message (for example hop count) in the best possible case to encourage the source node to employ this fake route. When the source node uses this fake route to send data, the attacker removes all data packets and does not allow any packet to reach the destination. A flooding node is identified based on the high sending rate of the RREQ messages for the target node. This behavior dramatically reduces the energy of the nodes and at the same time fills the memory of target nodes. This attacker knows that some information about these messages is kept in the target nodes, and when the memory of the nodes is overflowed, they are not able to respond to other nodes and will be removed from the network. Due to the limited energy of nodes, this attack has seriously damaged network lifetime and is a major challenge in WSN.

## Proposed scheme

**Figure 2:**
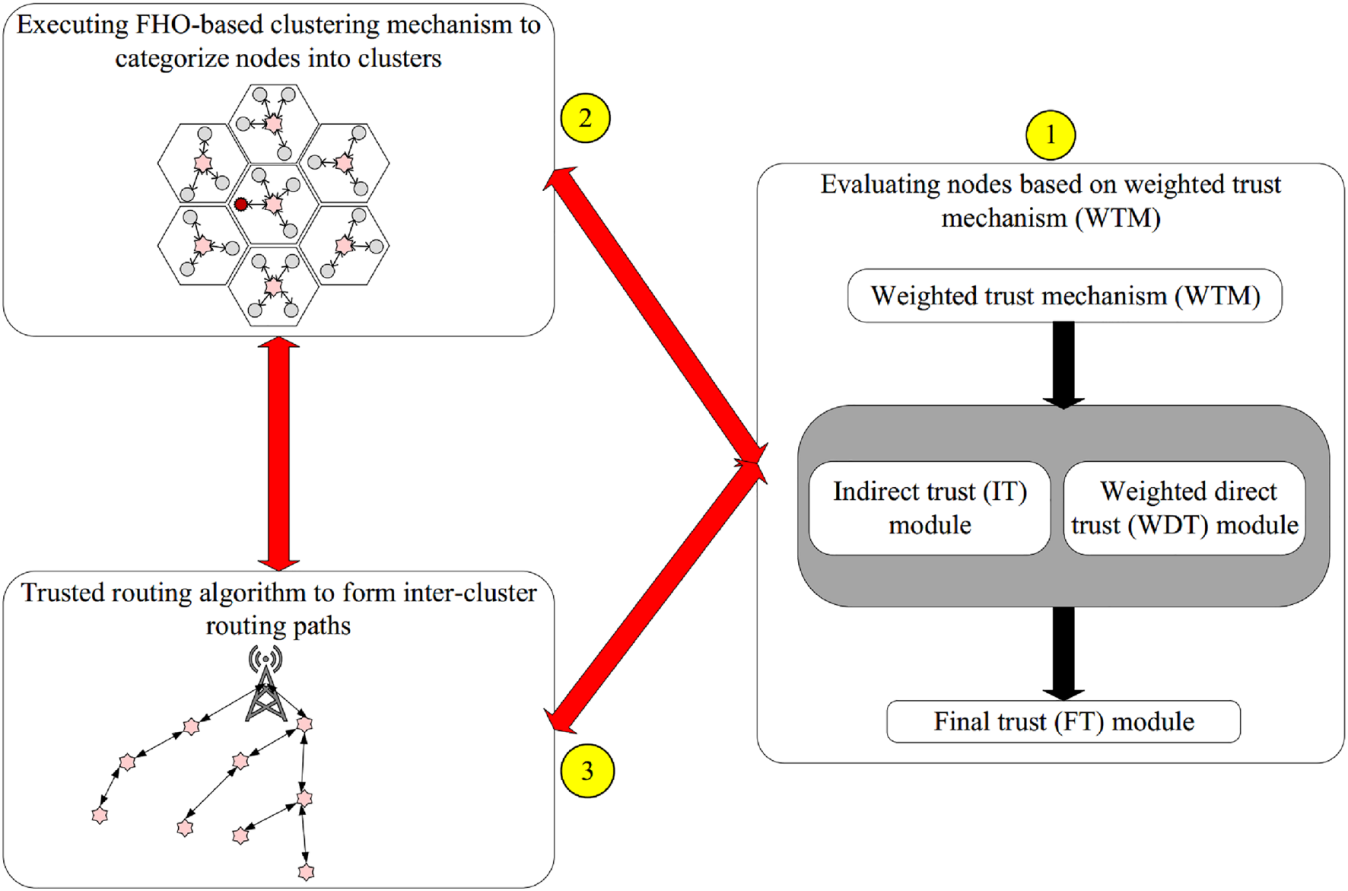
Diagram of the CTRF.

Here, the cluster-based trusted routing approach based on FHO (CTRF) is described. CTRF contains three main mechanisms: weighted trust mechanism (WTM), FHO-based clustering, and trusted routing. Figure 2 shows the diagram of the proposed method and the relationship between these three mechanisms. Also, Table 2 presents the most important notations used in this paper.

**Table 2:** Most important notations used in CTRF.

Notation	Description
$sn_{i}$	Sensor node *i*
*N*	Number of sensor nodes in the network
$ID_{sn_{i}}$	Identifier of $sn_{i}$
$E_{TX}$	Energy consumed by transmitter
$E_{RX}$	Energy consumed by receiver
${E_{elec}}$	Energy consumed by the electrical board of transmitter or receiver
${E_{fs}}$	Energy consumed by amplifier in the free space
${E_{mp}}$	Energy consumed by amplifier in the multi-path space
$WDT_{ij}^{t}$	Weighted direct trust of $sn_{i}$ relative to $sn_{j}$
$IT_{ij}^{t}$	Indirect trust of $sn_{i}$ relative to $sn_{j}$
$FT_{ij}^{t}$	Final trust of $sn_{i}$ calculated by $sn_{j}$
$WRR_{j}^{t}$	Weighted reception rate of $sn_{j}$ at a time interval $\left[ t-1,t\right]$
$PDR_{j}^{t}$	Packet delivery rate (PDR) of $sn_{j}$ at a time interval $\left[ t-1,t\right]$
$PK_{j}^{received}\left( t \right)$	Number of packets received by $sn_{j}$
$PK_{j}^{total}\left( t \right)$	All packets transferred to $sn_{j}$
$WRD_{j}^{t}$	Weighted redundancy rate of $sn_{j}$ at a time interval $\left[ t-1,t\right]$
$RD_{j}^{t}$	Redundancy rate of $sn_{j}$ at a time interval $\left[ t-1,t\right]$
$DupPK_{j}\left( t \right)$	Number of duplicate packets obtained from $sn_{j}$
$NewPK_{j}\left( t \right)$	Number of non-repeated packets obtained from $sn_{j}$
$ES_{j}^{t}$	Energy state of $sn_{j}$ at a time interval $\left[ t-1,t\right]$
$EC_{j}^{t}$	Total energy consumed by $sn_{j}$
$rn_{k}$	Recommended nodes
*R*	A set of all recommended nodes
*k*	Number of clusters in the network
$C_{j}$	Cluster *j*
$S_{i}$	Candidate solution *i*
*CCH*	A candidate CH set
$f_{cost}$	Cost function in the clustering mechanism
$CM_r$	Cluster member node *r*
$CH_j$	Cluster head node *j*
$FH_l$	Main fire in FHO
$PR_q$	*l*-th fire hawk in the search space
*GB*	*q*-th prey in the search space
$CH_s$	Source cluster head
$E_R$	Energy value of the route
$Q_R$	Quality of the path
$T_R$	Reliability of the path
$Route_k$	Route *k*
$H_c$	Number of hops in the path
$S_R$	Score of the route

### Weighted trust mechanism (WTM)

In CTRF, a weighted trust mechanism (WTM) is designed to estimate the trust of sensor nodes. It is a distributed trust mechanism. The main feature of WTM is to consider a regulatory coefficient for trust parameters. This coefficient reduces or increases the trust of sensor nodes in accordance with their hostile or friendly behaviors. In WTM, three main modules are defined: the weighted direct trust (WDT) module, the indirect trust (IT) module, and the final trust (FT) module. The pseudo-code related to WTM is stated in Algorithm 1.

#### WDT module

To distinguish between hostile nodes and normal nodes, $sn_{i}$ examines the behavioral pattern of its neighbor $sn_{j}$ to calculate WDT through this direct interaction. WDT is defined based on three weighted criteria.

***Criterion 1*** weighted reception rate ($WRR_{j}^{t}$): It is a weighted trust metric obtained from the packet delivery rate (PDR). At a specified time interval such as $\left[ t-1,t\right]$, $sn_{i}$ can calculate $PDR_{j}^{t}$ based on acknowledgments received by $sn_{j}$. In this regard, $sn_{i}$ uses Eq. (3) to calculate $PDR_{j}^{t}$.

3 $$\begin{aligned} PDR_{j}^{t}=\frac{PK_{j}^{received}\left( t \right) }{PK_{j}^{total}\left( t \right) }, \end{aligned}$$

where $PK_{j}^{received}\left( t \right)$ and $PK_{j}^{total}\left( t \right)$ are the number of packets received by $sn_{j}$ and all packets transferred to it, respectively.

Note that if $PDR_{j}^{t}$ is stable in different time intervals, this will increase the trust of $sn_{i}$ relative to $sn_{j}$ because it proves that the link between $sn_{i}$ and $sn_{j}$ is stable, meaning that, $sn_{j}$ is located in a stable situation. However, if $PDR_{j}^{t}$ fluctuates at different time intervals, meaning that it continuously increases or decreases, $sn_{j}$ is marked as a suspicious node, and the trust of $sn_{i}$ relative to $sn_{j}$ will be reduced. Therefore, the variance of $PDR_{j}^{t}$ (i.e. $var\left( PDR_{j}^{t}\right)$) is used to measure link stability. If $var\left( PDR_{j}^{t}\right)$ is close to zero, $PDR_{j}^{t}$ is stable. In contrast, if $var\left( PDR_{j}^{t}\right)$ is larger than zero, $PDR_{j}^{t}$ is more unstable. In general, the variance of a random variable is obtained from mathematical expectation ($E\left( X \right)$) according to Eq. (4).

4 $$\begin{aligned} var\left( X \right) =E\left( {X^{2}}\right) -{{\left( E\left( X \right) \right) }^{2}}. \end{aligned}$$

And

5 $$\begin{aligned} E\left( X \right) =\sum \limits _{i=1}^{n}{\frac{{X_{i}}}{n}}. \end{aligned}$$

Now, Eq. (6) combines Eqs. (5) and (4).

6 $$\begin{aligned} var\left( X \right) ={{\sum \limits _{i=1}^{n}{\frac{{{\left( {X_{i}}\right) }^{2}}}{n}-\left( \sum \limits _{i=1}^{n}{\frac{{X_{i}}}{n}}\right) }}^{2}}. \end{aligned}$$

As a result, $var\left( PDR_{j}^{t}\right)$ is calculated based on Eq. (7):

7 $$\begin{aligned} var\left( PDR_{j}^{t}\right) ={{\sum \limits _{x=1}^{{n_{PDR}}}{\frac{{{\left( PDR_{j}^{x} \right) }^{2}}}{{n_{PDR}}}-\left( \sum \limits _{x=1}^{{n_{PDR}}}{\frac{PDR_{j}^{x}}{{n_{PDR}}}}\right) }}^{2}}. \end{aligned}$$

Here, $n_{PDR}$ is the total number of sampled values of $PDR_{j}$, $PDR_{j}^{x}$ is *x*-th sampled value of $PDR_{j}$.

According to the mentioned points above, the weighted reception rate ($WRR_{j}^{t}$) is calculated from Eq. (8):

8 $$\begin{aligned} WPR_{j}^{t}=\left( PDR_{j}^{t}\right) {{e}^{-\left( \frac{\sqrt{var\left( PDR_{j}^{t}\right) }}{\sum \limits _{x=1}^{{{n}_{PDR}}}{\frac{PDR_{j}^{x}}{{n_{PDR}}}}}\right) }}. \end{aligned}$$

***Criterion 2*** weighted redundancy rate ($WRD_{j}^{t}$): It is a weighted trust metric calculated based on data redundancy. In WSN, redundancy (RD) is due to the wireless communication channels. Usually, the purpose of RD is to increase reliability and guarantee that data packets arrive at the desired node. Nevertheless, if the redundant packets exceeds a certain threshold, it is a suspicious event and the likelihood of a flooding attacker will be very high. At a specified time frame such as $\left[ t-1,t\right]$, $sn_{i}$ can calculate $RD_{j}^{t}$ based on Eq. (9).

9 $$\begin{aligned} RD_{j}^{t}=\frac{NewPK_{j}\left( t \right) }{NewPK_{j}\left( t \right) +DupPK_{j}\left( t \right) }, \end{aligned}$$

where $DupPK_{j}\left( t \right)$ and $NewPK_{j}\left( t \right)$ are the number of duplicate packets obtained from $sn_{j}$ and non-repeated packets at $\left[ t-1,t \right]$, respectively.

Now, if $sn_{i}$ receives a lot of duplicate packets from $sn_{j}$, it marks $sn_{j}$ as a suspicious node and reduces the trust relative to $sn_{j}$. Therefore, to penalize the nodes, which include high redundancy, a weight coefficient is added to $RD_{j}^{t}$ in Eq. (9) to obtain $WRD_{j}^{t}$ according to Eq. (10).

10 $$\begin{aligned} WRD_{j}^{t}=RD_{j}^{t}{e^{-\left( \frac{DupPK_{j}\left( t \right) }{NewPK_{j}\left( t \right) +DupPK_{j}\left( t \right) }\right) }}. \end{aligned}$$

***Criterion 3*** energy state ($ES_{j}^{t}$): In WSN, the energy consumed by normal nodes has a certain and stable level, while the energy consumed by flooding nodes is very high. As discussed in “Energy consumption model”, the energy used by sensor nodes for sending/receiving data is calculated in accordance with Eqs. (1) and (2), respectively. Therefore, the total energy consumed by $sn_{j}$ ($EC_{j}^{t}$) is equal to the sum of the consumed energy for sending and receiving packets in $\left[ t-1,t \right]$:

11 $$\begin{aligned} EC_{j}^{t}=\sum \limits _{x=1}^{{n_{EC}}}{\left( E_{TX}^{t}+E_{RX}^{t}\right) }, \end{aligned}$$

where $E_{TX}^{t}$ and $E_{RX}^{t}$ represent the energy consumed for sending and receiving data, respectively. Also, $n_{EC}$ indicates the number of data transfer operations carried out by $sn_{j}$ in $\left[ t-1,t\right]$. Therefore, the residual energy of $sn_{j}$ is equal to:

12 $$\begin{aligned} E_{res,j}^{t}=\left( \frac{E_{res,j}^{t-1}}{{E_{ini}}}\right) -\left( \frac{EC_{j}^{t}}{{E_{ini}}}\right) . \end{aligned}$$

Here, $E_{res,j}^{t-1}$ is the remaining energy of $sn_{j}$ in the moment $t-1$ and $E_{ini}$ represents the initial energy of the nodes.

Now, if the energy consumed of $sn_{j}$ in $\left[ t-1,t \right]$ is high, there is a high likelihood that $sn_{j}$ is a flooding node. Hence, the trust of $sn_{i}$ relative to $sn_{j}$ will be reduced. As a result, a coefficient is added to Eq. (12), and $ES_{j}^{t}$ is calculated based on Eq. (13).

13 $$\begin{aligned} ES_{j}^{t}=\left\{ \begin{matrix} 0,\,\,\,\,\,\,\,\,E_{res}^{t}\le 15\%\,\,\,\,of\,\,initial\,\,energy \\ E_{res}^{t}{{e}^{-\left( \frac{EC_{j}^{t}}{{E_{ini}}} \right) }},\,\,\,\,\,\,\,\,\,\,\,\,\,\,\,\,\,\,\,\,\,\,\,\,\,\,\,Otherwise \\ \end{matrix}\right. \end{aligned}$$

Finally, Eq. (14) estimates $WDT_{ij}^{t}$ based on the linear combination of these three criteria.

14 $$\begin{aligned} WDT_{ij}^{t}={{\lambda }_{1}}WRR_{j}^{t}+{{\lambda }_{2}}WRD_{j}^{t}+{{\lambda }_{3}}ES_{j}^{t}. \end{aligned}$$

Here, ${{\lambda }_{1}}$, ${{\lambda }_{2}}$, and ${{\lambda }_{3}}$ are weight coefficients in the interval $\left[ 0,1 \right]$, so that $\sum \nolimits _{i=1}^{3}{{{\lambda }_{i}}=1}$.

#### IT module

$sn_{i}$, in addition to considering its personal observations, uses the trust recommended by the recommended nodes ($rn_{k}$) to calculate the final trust. In WTM, $rn_{k}$ is the neighbor of both $sn_{i}$ and $sn_{j}$, and its trust level is higher than a threshold. Assume that $R=\left\{ rn_{1},rn_{2}, \ldots ,rn_{k},\ldots ,rn_{\left| R \right| }\right\}$ includes all recommended nodes between $sn_{i}$ and $sn_{j}$. In this case, $IT_{ij}^{t}$ is obtained from Eq. (15):

15 $$\begin{aligned} IT_{ij}^{t}=\frac{1}{\left| R \right| }\sum \limits _{k\in R}^{\left| R \right| }{\left( WDT_{ik}^{t}\cdot WDT_{kj}^{t}\right) }. \end{aligned}$$

So that $WDT_{ik}^{t}$ expresses the weighted direct trust of $sn_{i}$ relative to $rn_{k}$, $WDT_{kj}^{t}$ indicates the weighted direct trust of $rn_{k}$ relative to $sn_{j}$, *R* indicates a set of recommended nodes, and $\left| R \right|$ means the number of recommended nodes in *R*.

#### FT module

Finally, $sn_{i}$ gains its final trust relative to $sn_{j}$ (i.e. $FT_{ij}^{t}$) based on a linear combination of $WDT_{ij}^{t}$ and $IT_{ij}^{t}$. This is presented in Eq. (16).

16 $$\begin{aligned} FT_{ij}^{t}=\alpha WDT_{ij}^{t}+\left( 1-\alpha \right) IT_{ij}^{t}. \end{aligned}$$

So that $\alpha \in \left[ 0,1\right]$ expresses a regulatory factor. 
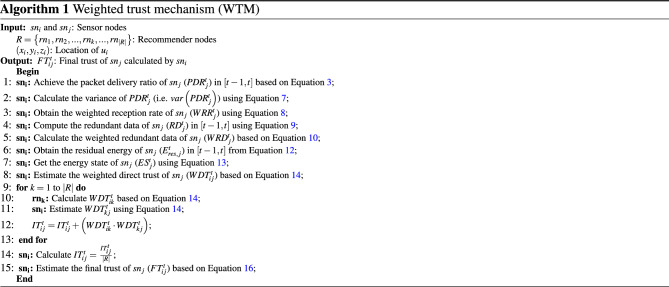


### FHO-based clustering mechanism

BS has the responsibility to design and execute the FHO-based clustering mechanism. In this mechanism, we assume that BS monitors network nodes ($sn_{i}$, where $i=1,2,\ldots ,N$) and is aware of their status (i.e. trust, position, and energy). This information is obtained by the regular exchange of guide messages between nodes and BS. In the clustering mechanism, the second assumption is that the number of clusters is predetermined (*k* clusters) so that the clusters are displayed as ${C_{1},C_{2},\ldots ,C_{k}}$. Furthermore, the role of the cluster head changes rotationally between the nodes to prevent the discharge of the nodes and balance the consumed energy in the network. Hence, all nodes can be cluster heads. In each period, BS uses the FHO algorithm to select the best CHs in the network. The pseudo-code of this mechanism is stated in Algorithm 2. The different steps of the clustering mechanism are as follows:

- *Step (1) Initialization operation of population* In this step, BS considers candidate solutions ($S_{i}$), which are corresponding to fire hawks and prey. In the CH selection problem, each fire hawk or prey is considered an array with *k* elements (So that *k* indicates the number of CHs). In this array, each element includes the ID of a sensor node (such as $sn_{j}$). This ID is randomly selected from a candidate CH set called *CCH*. This set is defined in Eq. (17):
  17 $$\begin{aligned} CCH=\left\{ sn_{j}|E_{res,j}^{t}\ge \frac{\left( \sum \nolimits _{i=1}^{N}{E_{res,i}^{t}} \right) }{N},FT_{j}^{t}\ge \frac{\left( \sum \nolimits _{i=1}^{N}{FT_{i}^{t}}\right) }{N}\right\} . \end{aligned}$$
  Note that the *CCH* set includes the ID of the sensor nodes whose remaining energy ($E_{res,j}^{t}$) and trust level ($FT_{j}^{t}$) are more than the average remaining energy and the average trust level of all network nodes, respectively. As a result, low-energy nodes and insecure nodes cannot be selected as CH. This process is stated in Eq. (18).
  18 $$\begin{aligned} S=\left[ \begin{array}{ll} \begin{array}{ll} {S_{1}} \\ {S_{2}} \\ \,\vdots \\ \end{array} \\ \begin{array}{ll} {S_{i}} \\ \,\vdots \\ {S_{P}} \\ \end{array} \\ \end{array} \right] =\left[ \begin{array}{ll} \begin{array}{ll} s_{1}^{1},s_{1}^{2},\ldots ,s_{1}^{j},\ldots ,s_{1}^{k} \\ s_{2}^{1},s_{2}^{2},\ldots ,s_{2}^{j},\ldots ,s_{2}^{k} \\ \qquad \qquad \vdots \\ \end{array} \\ \begin{array}{ll} s_{i}^{1},s_{i}^{2},\ldots ,s_{i}^{j},\ldots ,s_{i}^{k} \\ \qquad \qquad \vdots \\ s_{P}^{1},s_{P}^{2},\ldots ,s_{P}^{j},\ldots ,s_{P}^{k} \\ \end{array} \\ \end{array} \right] , \,\,\,\,\,\,\left\{ \begin{array}{ll} &{}i=1,2,\ldots ,P \\ &{}j=1,2,\ldots ,k \\ \end{array} \right. \end{aligned}$$
  Here, $S_{i}$ refers to the *i*-th candidate solution in the search area. *k* is the number of CHs. *P* indicates the total number of candidate solutions in the CH selection problem. Moreover, $s_{i}^{j}$ represents the ID of $sn_{j}$ that is randomly selected from the *CCH* set and inserted in the candidate solution $S_{i}$.
- *Step (2) Evaluation process* In this step, each candidate solution is evaluated according to the cost function presented in Eq. (19).
  19 $$\begin{aligned} f_{cost}=\sum \limits _{i=1}^{4}{{{\omega }_{i}}{f_{i}}}, \end{aligned}$$
  where ${{\omega }_{i}}\in \left[ 0,1 \right]$ are weight coefficients and $\sum \nolimits _{i=1}^{4}{{{\omega }_{i}}=1}$.
  Given that $f_{cost}$ is a cost function, thus, an optimal solution is achieved when $f_{cost}$ is minimized. In Eq. (19), $f_{cost}$ is a linear combination of $f_{1}$, $f_{2}$, $f_{3}$, and $f_{4}$.
  According to $f_{1}$ in Eq. (20), BS prefers to select nodes as CH, which satisfy two conditions: (1) These sensor nodes are nearest to the cluster center, meaning that the distance between cluster member nodes ($CM_{r}\in {C_{j}}$) and the corresponding CH ($CH_{j}$) is minimized. (2) Distance between CHs must be high so that CHs are well distributed in all areas of the network.
  20 $$\begin{aligned} f_{1}=\frac{\sum \nolimits _{j=1}^{k}{\left( \frac{\sum \nolimits _{\forall \,CM_{r}\in {C_{j}}}{d\left( CM_{r},CH_{j}\right) }}{\left| {C_{j}}\right| }\right) }}{\underset{\forall \,\,CH_{j}\ne CH_{g}}{\mathop {\min }}\,\left\{ d\left( CH_{j},CH_{g}\right) \right\} }, \end{aligned}$$
  where $\left| {C_{j}}\right|$ is the size of the cluster $C_{j}$, $d\left( CM_{r},CH_{j} \right) =\sqrt{{{\left( {x_{r}}-{x_{j}}\right) }^{2}}+{{\left( {y_{r}}-{y_{j}}\right) }^{2}}}$ represents the Euclidean distance of $CM_{r}$ with spatial coordinates $\left( {x_{r}},{y_{r}}\right)$ and $CH_{j}$ with spatial coordinates $\left( {x_{j}},{y_{j}}\right)$. Also, $d\left( CH_{j},CH_{g}\right)$ is the distance between $CH_{j}$ and $CH_{g}$.
  On the other hand, based on $f_{2}$ in Eq. (21), BS prefers to select CHs from high-energy nodes because $f_{2}$ is the sum of the ratio of the average energy of CMs to the energy of CHs. To minimize $f_{2}$, the average remaining energy of CMs must be less than the residual energy of CHs.
  21 $$\begin{aligned} f_{2}=\sum \limits _{j=1}^{k}{\left( \frac{\left( \frac{\sum \nolimits _{\forall \,CM_{r}\in C_{j}}{E_{res,r}^{t}}}{\left| {C_{j}}\right| }\right) }{E_{res,j}^{t}}\right) }, \end{aligned}$$
  where $E_{res,r}^{t}$ and $E_{res,j}^{t}$ are the residual energies of $CM_{r}$ and $CH_{j}$, respectively. Also, according to $f_{3}$ in Eq. (22), BS prefers to select CHs from nodes, which are nearest to BS. This decreases delay and energy used when transferring data between CHs and BS.
  22 $$\begin{aligned} f_{3}=\underset{j-1,2, \ldots ,k}{\mathop {\max }}\,\left\{ d\left( CH_{j},BS\right) \right\} . \end{aligned}$$
  According to $f_{4}$ in Eq. (23), BS prefers that the size of all clusters is almost equal to each other. Therefore, the standard deviation can be used to compare the size of clusters. If this metric is close to zero, the size of the clusters will be almost equal to each other.
  23 $$\begin{aligned} f_{4}=\underset{j=1,2,\ldots ,k}{\mathop {\max }}\,\left\{ \frac{\sqrt{\sum \nolimits _{j=1}^{k}{{{\left( \left| {C_{j}}\right| -\left( \frac{\sum \nolimits _{j=1}^{k}{\left| {C_{j}}\right| }}{k}\right) \right) }^{2}}}}}{\left( \frac{\sum \nolimits _{j=1}^{k}{\left| {C_{j}}\right| }}{k}\right) }\right\} . \end{aligned}$$
  After evaluating the solutions, the best solution is specified as the main fire (*GB*). Then, other candidate solutions will be categorized into two classes according to the cost value: fire hawk and prey, so that the solutions with less cost function are fire hawks (Eq. 24) and other solutions are considered prey (Eq. 25).
  24 $$\begin{aligned} FH=\left[ \begin{array}{ll} \begin{array}{ll} FH_{1} \\ FH_{2} \\ \,\,\vdots \\ \end{array} \\ \begin{array}{ll} FH_{l} \\ \,\,\vdots \\ FH_{f} \\ \end{array} \\ \end{array} \right] ,l=1,2, \ldots ,f. \end{aligned}$$
  25 $$\begin{aligned} PR=\left[ \begin{array}{ll} \begin{array}{ll} PR_{1} \\ PR_{2} \\ \,\,\vdots \\ \end{array} \\ \begin{array}{ll} PR_{q} \\ \,\,\vdots \\ PR_{m} \\ \end{array} \\ \end{array} \right] ,q=1,2, \ldots ,m. \end{aligned}$$
  So that $FH_{l}$ is the *l*-th fire hawk, *f* defines the number of fire hawks. $PR_{q}$ indicates the *q*-th prey in the search space, and *m* indicates the number of prey.
- *Step (3) Determining the territory of fire hawks* In this step, each fire hawk determines preys close to itself as its territory. To determine the territory of each fire hawk, the sum of the Euclidean distance between the selected CHs in $PR_{q}$ and the selected CHs in $FH_{l}$ is calculated using Eq. (26).
  26 $$\begin{aligned} D_{q}^{l}=\sum \limits _{l=1}^{f}{\sum \limits _{q=1}^{m}{\sqrt{\sum \limits _{j=1}^{k}{{{\left( s_{l}^{j}-s_{q}^{j}\right) }^{2}}}}}}. \end{aligned}$$
  $s_{l}^{j}$ and $s_{q}^{j}$ are the *j*-th CH in the *l*-th fire hawk and the *j*-th CH in the *q*-th prey, respectively.
- *Step (4) Updating fire hawks* In this step, each fire hawk gets burning woods from *GB* and sets fire in its territory to pressurize prey to flee. This behavior is used to refresh the position of the fire hawk ($FH_{l}^{new}=\left[ \tilde{s}_{l}^{1},\tilde{s}_{l}^{2}, \ldots ,\tilde{s}_{l}^{k}\right]$) in accordance with Eq. (27).
  27 $$\begin{aligned} \tilde{s}_{l}^{i}=\left\{ \begin{matrix} s_{l}^{j},\,\,\,\,\,\,\,\,\,\,\,\,\,\,\,\,\,\,\,\,\,\,{r_{1}}=0\,and\,{r_{2}}=0 \\ s_{near-to-GB}^{j},\,\,{r_{1}}\ne 0\,and\,{r_{2}}<0.5 \\ s_{near-to-betterFH}^{j},\,\,\,\,\,\,\,\,\,\,\,Otherwise \\ \end{matrix} \right. ,\left\{ \begin{matrix} j=1,2, \ldots ,k \\ l=1,2,\ldots ,f \\ \end{matrix} \right. \end{aligned}$$

Here, $s_{near-to-GB}^{j}$ means the selection of a node from the *CCH* set so that this node is closer to the corresponding CH in *GB*. Moreover, $s_{near-to-betterFH}^{j}$ refers to the selection of a node from the *CCH* set so that this node is close to the corresponding CH in the fire hawk with less cost function than the current fire hawk. $r_{1}$ and $r_{2}$ are random numbers in $\left[ 0,1 \right]$.

- *Step (5) Updating prey* In this step, when the fire hawk releases a burning wood in its territory, the prey must decide to adjust its movement in the search area. This decision is applied to calculate the new position of the prey (i.e. $PR_{q}^{new}=\left[ \tilde{s}_{q}^{1},\tilde{s}_{q}^{2},\ldots ,\tilde{s}_{q}^{k}\right]$). Each element of $PR_{q}^{new}$ is obtained using Eq. (28).
  28 $$\begin{aligned} \tilde{s}_{q}^{i}=\left\{ \begin{matrix} s_{q}^{j},\,\,\,\,\,\,\,\,\,\,\,\,\,\,\,\,\,\,\,{r_{3}}=0\,\,and\,\,{r_{4}}=0 \\ s_{near-to-FH}^{j}\,{r_{3}}\ne 0\,\,and\,\,{r_{4}}<0.5 \\ s_{random}^{j},\,\,\,\,\,\,\,\,\,\,\,\,\,\,\,\,\,\,\,\,\,\,\,\,\,\,Otherwise \\ \end{matrix}\right. \end{aligned}$$
  So that $s_{near-to-FH}^{j}$ means the selection of a node from the *CCH* set so that this node is closer to the position of corresponding CH in the fire hawk related to the prey. $s_{random}^{j}$ refers to the selection of a random node from the *CCH* set. $r_{3}$ and $r_{4}$ are two random numbers in $\left[ 0,1 \right]$.
  Then, $PR_{q}^{new}$ evaluates using the cost function in Eq. (19). If the updated prey cannot improve the cost function compared to the previous one, $PR_{q}^{new}$ is re-calculated based on Eq. (26) because the prey may move toward the territory of other fire hawks.
  29 $$\begin{aligned} \tilde{s}_{q}^{i}=\left\{ \begin{matrix} s_{q}^{j},\,\,\,\,\,\,\,\,\,\,\,\,\,\,\,\,\,\,\,\,\,\,\,\,\,\,{r_{5}}=0\,\,and\,\,{r_{6}}=0 \\ s_{near-to-alterFH}^{j}\,{r_{3}}\ne 0\,\,and\,\,{r_{4}}<0.5 \\ s_{random}^{j},\,\,\,\,\,\,\,\,\,\,\,\,\,\,\,\,\,\,\,\,\,\,\,\,\,\,\,\,\,\,\,\,Otherwise \\ \end{matrix}\right. \end{aligned}$$
  Here, $s_{near-to-alterFH}^{j}$ means the selection of a node from the *CCH* set so that this node is close to the position of the corresponding CH in a fire hawk. $r_{5}$ and $r_{6}$ are two random numbers in $\left[ 0,1 \right]$.
- *Step (6) Convergence condition* This step expresses the end condition of the FHO algorithm. If the end condition is met, FHO will be finished, and *GB* is returned as the final solution. In the clustering mechanism, the stop condition is $\lambda$ iterations so that $\lambda >0$. After completing the algorithm, BS sends a state determination (SD) message to the network nodes and specifies their status as CH or CM. After forming clusters, the data transmission phase is started so that CMs will send their data directly to CH according to the determined scheduling. As soon as CHs receive the data from CMs, they aggregate this data and forward the merged data to BS through the paths specified in “A trusted inter-cluster routing mechanism”.

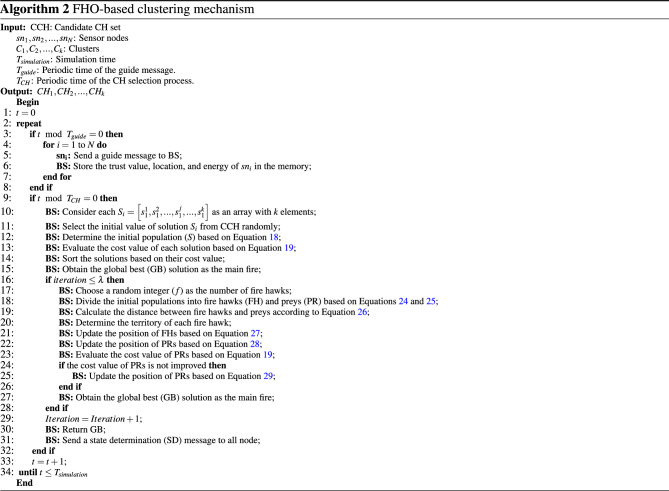


### A trusted inter-cluster routing mechanism

This section introduces an on demand routing technique, meaning that if $CH_{S}$ wants to transfer data packets to BS, it will search the routing paths. There are two modes in this problem:

- *Mode 1* If $CH_{S}$ and BS are neighbors, $CH_{S}$ transmits its data directly to BS.
- *Mode 2* If $CH_{S}$ and BSs are not neighbors, $CH_{S}$ must find a valid path to BS.

**Figure 3:**
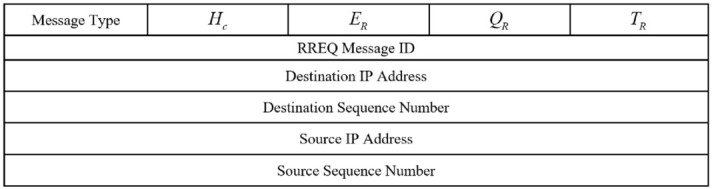
RREQ format.

To achieve this goal, $CH_{S}$ makes a route request (RREQ) message and transfers this message to its neighboring CHs. According to Fig. 3, the format of RREQ in our routing mechanism is similar to that in AODV, but there are three differences.

- ${{\textbf{E}}_{\textbf{R}}}$: This field maintains the energy value of the route. This metric equals the lowest residual energy of the nodes in this path.
  30 $$\begin{aligned} E_{R}=\underset{\forall \,\,sn_{i}\in Route}{\mathop {\min }}\,\left\{ E_{res,i}^{t}\right\} . \end{aligned}$$
  Here, $E_{res,i}^{t}$ is the residual energy of $sn_{i}$ in the route.
- ${\mathbf {Q_{R}}}$: This field stores the quality of the path creates between $CH_{S}$ and BS. It is dependent on the quality of the links available in the path. In the routing process, the quality of the link between $CH_{i}$ and $CH_{j}$ is obtained from the ratio of the packets obtained from $CH_{j}$ to packets sent by $CH_{i}$. The number of packets obtained from $CH_{j}$ is equal to the number of ACK obtained from $CH_{i}$. Therefore, $CH_{i}$ can estimate the quality of the link between itself and $CH_{j}$ in a specific time interval $\left[ t-1,t\right]$ based on Eq. (31).
  31 $$\begin{aligned} Q_{ij}=\frac{PK_{j}^{received}\left( t \right) }{PK_{j}^{total}\left( t \right) }, \end{aligned}$$
  where $PK_{j}^{received}\left( t \right)$ and $PK_{j}^{total}\left( t \right)$ are the number of packets received by $CH_{j}$ and all packets transmitted to it in $\left[ t-1,t\right]$, respectively. Therefore, $Q_{R}$ is equal to the minimum link quality in the route.
  32 $$\begin{aligned} Q_{R}=\underset{\forall \,\,sn_{i},sn_{j}\in Route}{\mathop {\min }}\,\left\{ {Q_{ij}}\right\} . \end{aligned}$$
- ${\mathbf {T_{R}}}$: This field represents the reliability of the path. The amount of this field is equal to the minimum trust level in the path.
  33 $$\begin{aligned} T_{R}=\underset{\forall \,\,CH_{i},CH_{j}\in Route_{k}}{\mathop {\min }}\,\left( FT_{ij}^{t}\right) , \end{aligned}$$
  where $CH_{i}$ and $CH_{j}$ indicate the previous-hop node and the current node in $Route_{k}$, respectively. Moreover, $FT_{ij}^{t}$ indicates the trust level of $CH_{i}$ relative to $CH_{j}$. It is explained in “Weighted trust mechanism (WTM)”.

After receiving RREQ, each node controls its ID to ensure that this message is new. Then, RREQ will be re-broadcast until it is received by BS. Now, BS uses Eq. (34) to calculate the score of all discovered paths based on the information inserted into RREQs.

34 $$\begin{aligned} S_{R}=\frac{{T_{R}}+{E_{R}}+{Q_{R}}}{{H_{c}}}. \end{aligned}$$

So that $E_{R}$, $Q_{R}$, $T_{R}$, and $H_{c}$ are the route energy, the route quality, the route reliability, and hop count in the path, respectively.

Finally, BS picks out a path with the most score and sends a route reply (RREP) message to $CH_{S}$ through this path. After creating this route, $CH_{S}$ will use it to transfer its data to BS. Note that the route maintenance operation in CTRF is similar to that in AODV. The pseudo-code related to this mechanism is stated in Algorithm 3. 
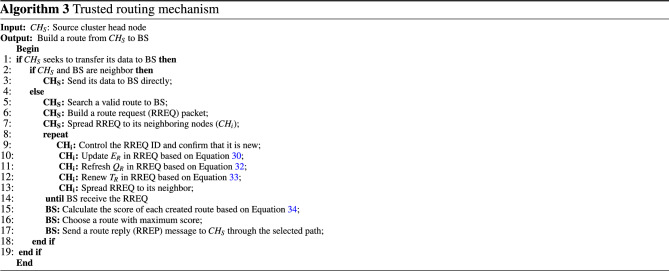


## Simulation and result evaluation

Here, the simulation process of CTRF is performed through NS2, and the results are evaluated in accordance with consumed energy, detection rate of hostile nodes, accuracy, throughput, packet loss rate, and delay. Then, these results are compared with those of 3LWT-GWO^21^, SASR^23^, and AF-TNS^22^. In this process, the network size equals $100\times 100\,\,m^{2}$, and it contains 100 nodes. Each sensor node has the initial energy (i.e. 1 J) and its communication radius is 30 meters. These sensors consume energy equal to 0.01 J to send/receive each bit. In the simulation operation, the data packet is 512 bytes, and the runtime equals 500 seconds. Additionally, it is assumed that the number of hostile nodes changes between 10 and 50% of the network nodes. The location of BS is fixed and equal to $\left( 100,10\right)$. Table 3 summarizes the most important parameters used in the simulation process.

**Table 3:** Simulation settings.

Parameter	Value
Simulation tool	NS2
The dimensions of network	$100\times 100\,\,m^{2}$
BS position	$\left( 50,100 \right)$
Attack	Flooding
Data packet	512 Bytes
The number of sensor nodes	100
The number of attackers	10–50%
Primary energy of nodes	1 J
Connection radius of nodes	30 m
Location of BS	$\left( 100,10 \right)$
Energy required for sending/receiving each bit	0.01 J
$E_{mp}$	$0.0013\,\,\text{pJ/bit/m}^{2}$
$E_{fs}$	$10\,\, \text{pJ/bit/m}^{2}$
$E_{elec}$	50 nJ/bit

### Energy

**Figure 4:**
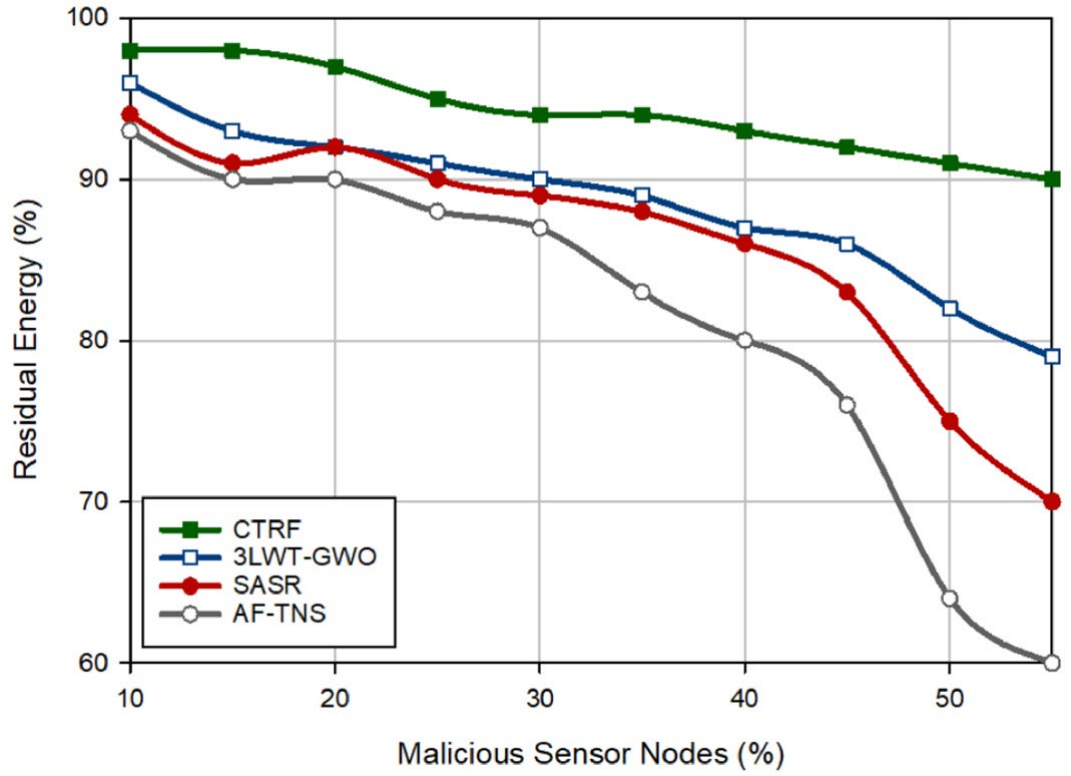
Comparison of residual energy in different approaches.

**Figure 5:**
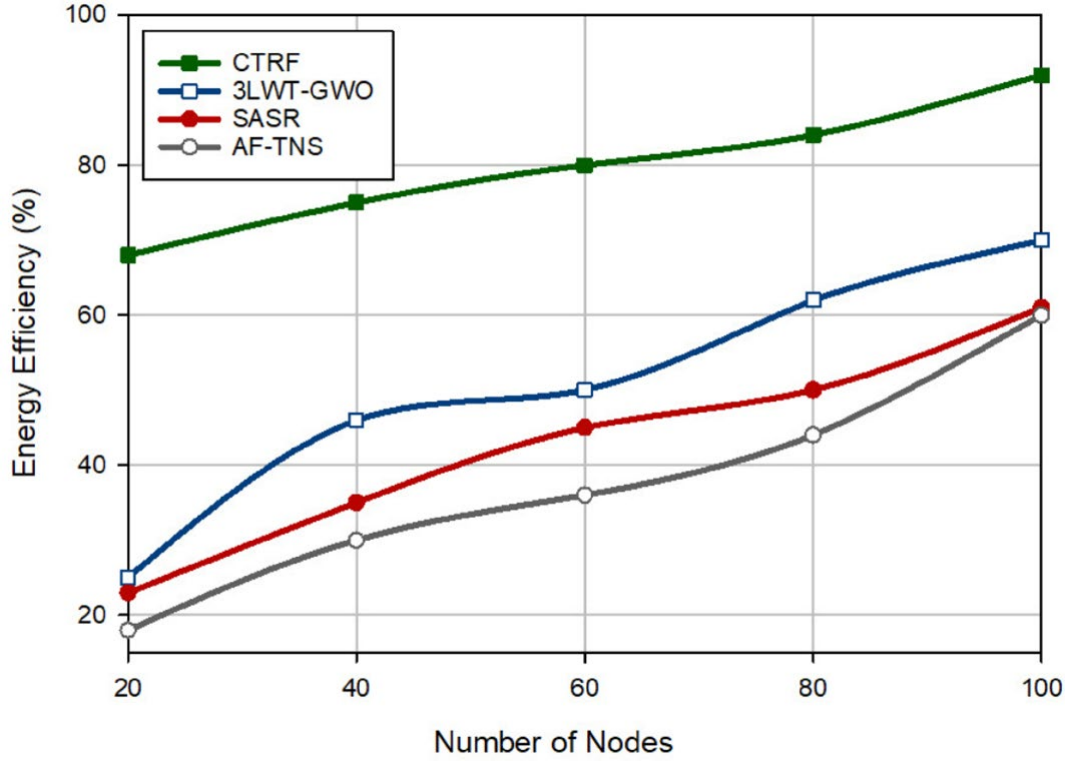
Comparison of energy efficiency in different approaches.

Figure 4 analyzes the remaining energy in different protocols. Note that the energy consumed in each node is equal to the sum of the required energy to perform the data transmission operation (sending/receiving data). As shown in Fig. 4, CTRF has the best residual energy level and improves this factor by 6.44%, 9.79%, and 16.15% compared to 3LWT-GWO, SASR, and AF-TNS, respectively. This is because CTRF regards three parameters, including weighted reception rate, weighted redundant rate, and consumed energy for designing the weighted trust mechanism (WTM). This trust mechanism considers the exponential coefficients for these trust parameters to decrease the trust level of hostile nodes rapidly based on their hostile behavior. As a result, WTM identifies hostile nodes well and prevents them from misbehaving in the network. As shown in Fig. 4, WTM reduces the destructive effect of hostile nodes on the energy level of nodes and improves energy consumption in CTRF. On the other hand, our scheme pays attention to the energy of the discovered paths in the routing mechanism and considers the energy of nodes in the clustering process. These mentioned points have a positive effect on CTRF performance. In addition, according to Fig. 4, there is an opposite relation between the number of attackers and the residual energy level. If the network contains a lot of attackers, the energy level in all protocols will decrease, and vice versa because one of the negative effects of malicious nodes is to rise the energy consumed by the target nodes when transferring the large number of RREQs on the network. In addition, Fig. 5 shows energy efficiency in different schemes. According to this figure, CTRF has the best energy efficiency and increases it by 57.71%, 86.45%, and two times compared to 3LWT-GWO, SASR, and AF-TNS, respectively. This proves that CTRF can extend network lifetime. Figure 5 shows when the number of sensor nodes is increasing, energy efficiency is also rising. This means that these two parameters have a direct relationship with each other. The reason for this issue is quite clear. When the density of the network is high, the distance between the nodes becomes shorter, as a result, the sensor nodes can find better and more stable routes between themselves, and its result is an increase in energy efficiency in the network.

### Throughput

**Figure 6:**
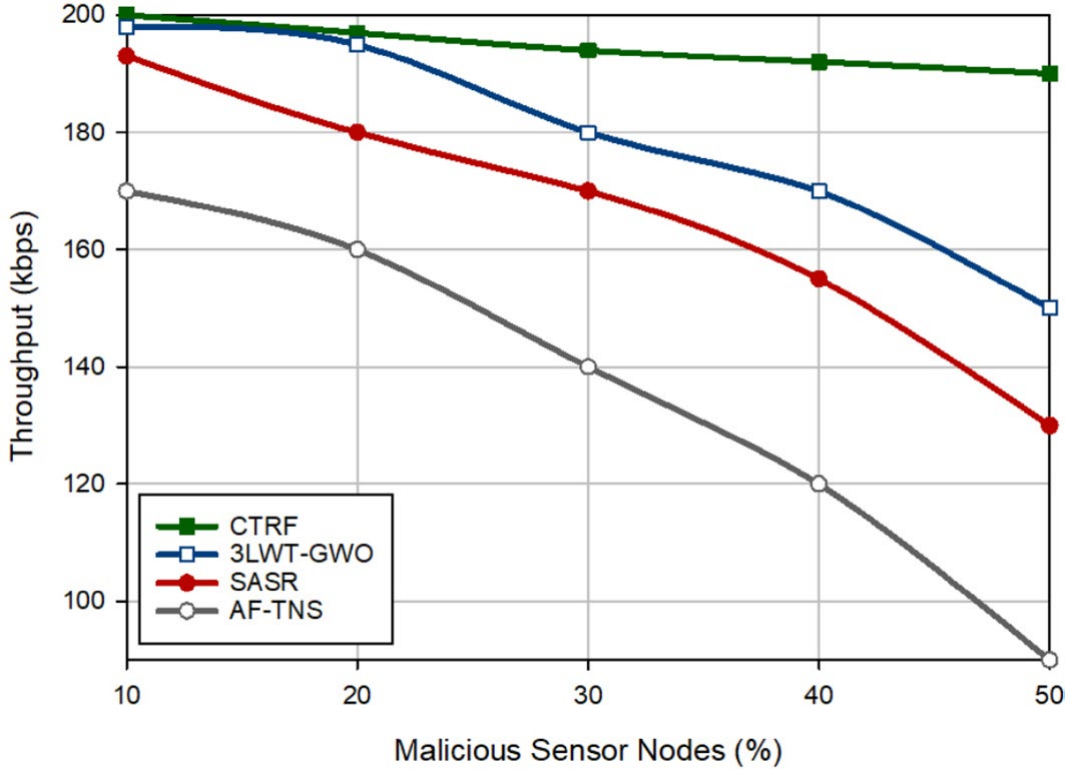
Evaluation of throughput in the routing schemes.

In Fig. 6, throughput is compared in different schemes. Throughput means the number of packets delivered to the destination at a given time interval. CTRF has the highest efficiency compared to other approaches and enhances it by 8.96%, 17.51%, and 43.09%, respectively. This is because CTRF considers the quality of the discovered paths when selecting the best path to the destination. Thus, CTRF can increase the data delivery rate, which has a positive effect on throughput. Furthermore, CHs are chosen among trusted nodes to prevent the negative effect of malicious nodes on intra-cluster and inter-cluster communications. According to Fig. 6, it can be found that there is a reverse relationship between throughput and the number of attackers, meaning that if the network contains a lot of attackers, throughput in different methods will be reduced. In Fig. 6, CTRF is less affected by malicious nodes because the security mechanism designed in this method can well detect and isolate the malicious nodes. Hence, they cannot have a negative effect on the performance of this scheme.

### Packet loss rate (PLR)

**Figure 7:**
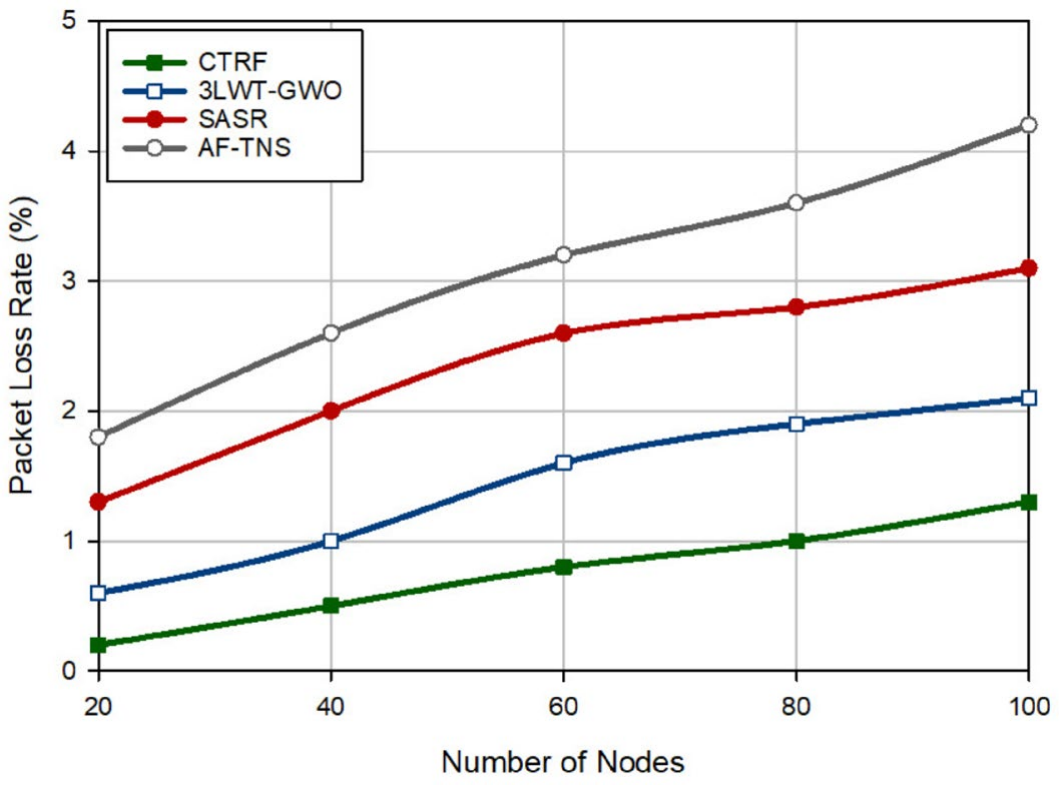
Evaluation of packet loss rate in different methods.

In Fig. 7, packet loss rate (PLR) in different methods is compared with each other. PLR means the ratio of lost packets to total packets sent to the destination. According to Fig. 7, CTRF has the lowest PLR and reduces it by 47.22%, 60.80%, and 75.32% compared to 3LWT-GWO, SASR, and AF-TNS, respectively. The successful performance of CTRF in PLR is due to its strong trust mechanism (i.e. WTM). This mechanism is strong because WTM continuously checks the behavior of nodes in the routing process. If these nodes do not have a good packet delivery rate or broadcast a large number of duplicate packets in the network, these nodes are suspicious, and WTM quickly reduces their trust based on exponential coefficients. As a result, WTM quickly and timely identifies and isolates hostile nodes in the network. This causes hostile nodes to be excluded from participating in the routing process. Therefore, in the path selection process, a normal node seeks to find a path that does not pass through these untrusted nodes. This problem has a positive effect on reducing the number of lost packets in the network. On the other hand, the path selection process in CTRF uses a parameter called path quality to find the best path in the data transmission process. Path quality evaluates the packet delivery rate at intermediate nodes in a path. Consequently, if a path has poor quality, it is not selected for data transmission. The reasons mentioned above reduce PLR in CTRF. On the other hand, according to Fig. 7, when the number of nodes in the network is increasing, PLR also has an upward trend, its main reason is that the congestion in the network increases and consequently some packets will be lost due to collision.

### Delay

**Figure 8:**
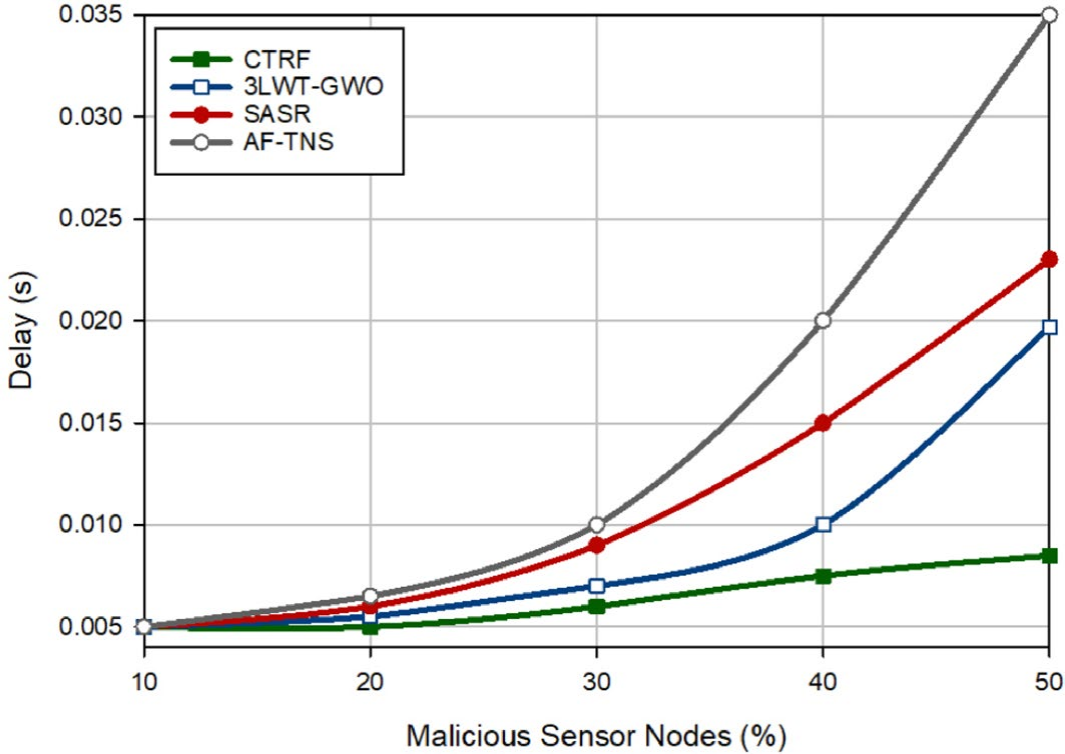
Evaluation of delay in different approaches.

Figure 8 compares delay in different approaches. Delay means the average spent time to forward a data packet from source to destination. CTRF reduces delay by 32.20%, 42.83%, and 58.17% compared to 3LWT-GWO, SASR, and AF-TNS. The main reason for this is that CTRF selects high-energy, high quality, and reliable paths for the data transfer process. As a result, this reduces route failure and hence, the need for the route discovery process, which is a delayed process, will be reduced. Moreover, according to Fig. 8, delay and the number of attackers have an opposite relation and when the network contains a lot of attackers, the network delay is also high. In CTRF, the trust mechanism can identify and separate hostile nodes quickly and timely because WTM considers exponential coefficients for the trust parameters, namely weighted reception rate, weighted redundancy rate, and energy state and decreases the trust levels of hostile nodes rapidly based on their hostile behavior. In the clustering process, the nodes whose trust levels are lower than the average trust level of all network nodes cannot be selected as the cluster head. As a result, clusters are managed by secure CHs. In addition, adversarial nodes cannot act as intermediate nodes in a route because the path selection process takes into account the reliability of the path, consequently, and routes that include adversarial nodes are not used for the data transmission process. These reasons have caused CTRF to perform successfully in terms of delay in the network in the presence of hostile nodes.

### Detection rate

**Figure 9:**
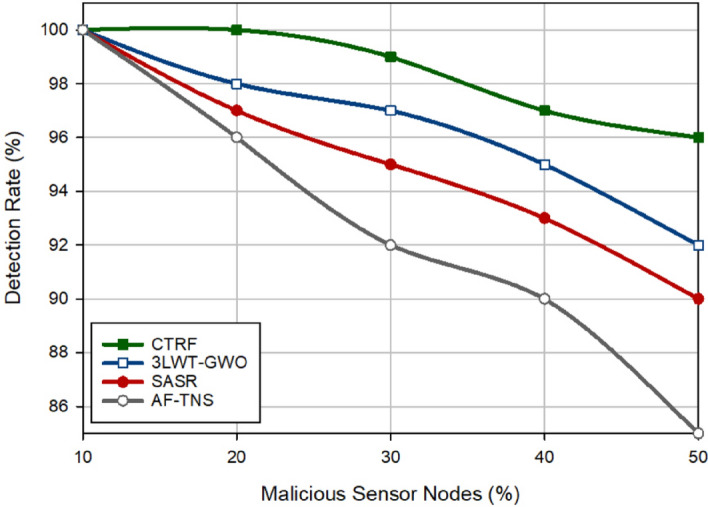
Detection rate in different approaches.

**Figure 10:**
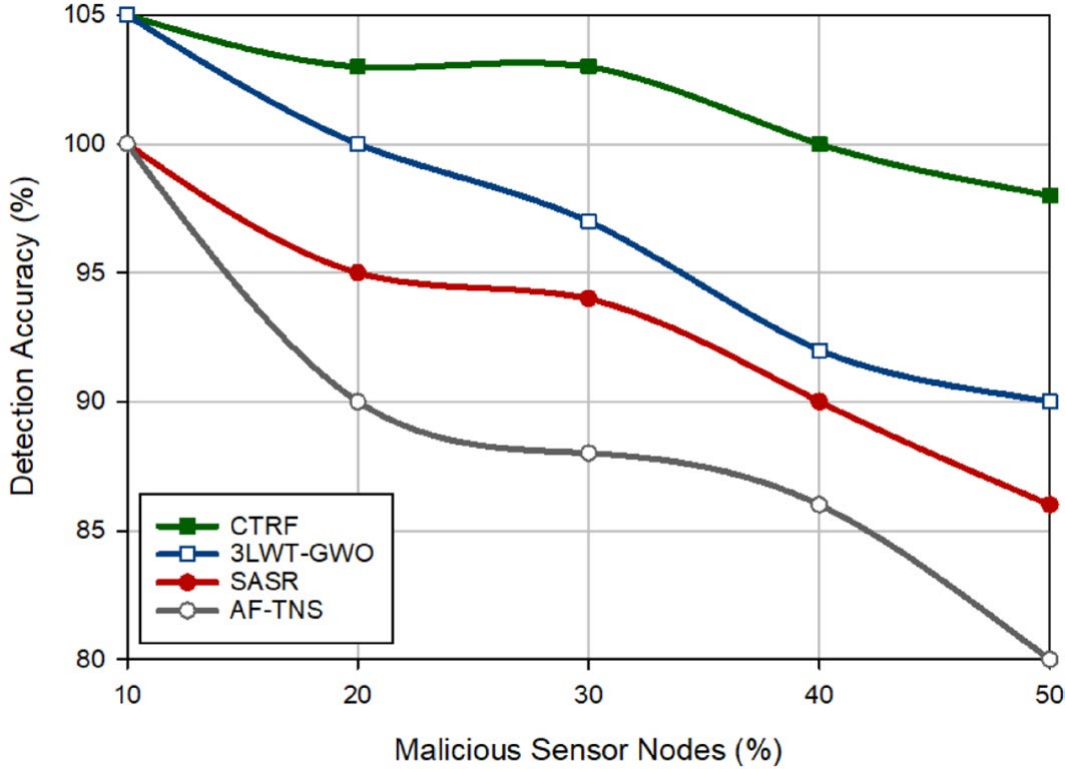
Accuracy in different approaches.

In Fig. 9, the detection rate has been compared in different approaches. The detection rate indicates the strength of the trust system designed in various schemes to correctly detect malicious nodes on the network. It is equal to the ratio of the detected malicious nodes to all malicious nodes in the network. CTRF improves the detection rate by 2.07%, 3.58%, and 6.26% compared to 3LWT-GWO, SASR, and AF-TNS. The main reason for the suitable detection strength of CTRF is that the weighted trust mechanism regularly monitors the behavior of sensor nodes in the network and checks their trust parameters. If the packet delivery rate of a sensor node changes frequently, WTM detects this node as a black hole attacker and decreases its trust level exponentially. Furthermore, if a sensor node experiences a high redundancy rate or its energy level changes highly, WTM identifies this node as a flooding node and reduces the corresponding trust parameters based on an exponential coefficient. Therefore, these weight coefficients have increased the capability of WTM in detecting hostile behaviors of attackers. As shown in Fig. 9, the detection rate and the number of attackers have a contradictory relationship and when the network contains a lot of attackers, their detection will be more difficult for security systems because these nodes can collude with each other and hide in the network. Figure 10 displays the detection accuracy of these routing approaches. Accuracy indicates the relationship between the real results and the results predicted by the trust systems. According to Fig. 10, the proposed scheme increases accuracy by 5.11%, 9.46%, and 14.64% compared to 3LWT-GWO, SASR, and AF-TNS, respectively. This shows that the proposed weighted trust system has a high accuracy for detecting malicious nodes.

### Communication cost

**Figure 11:**
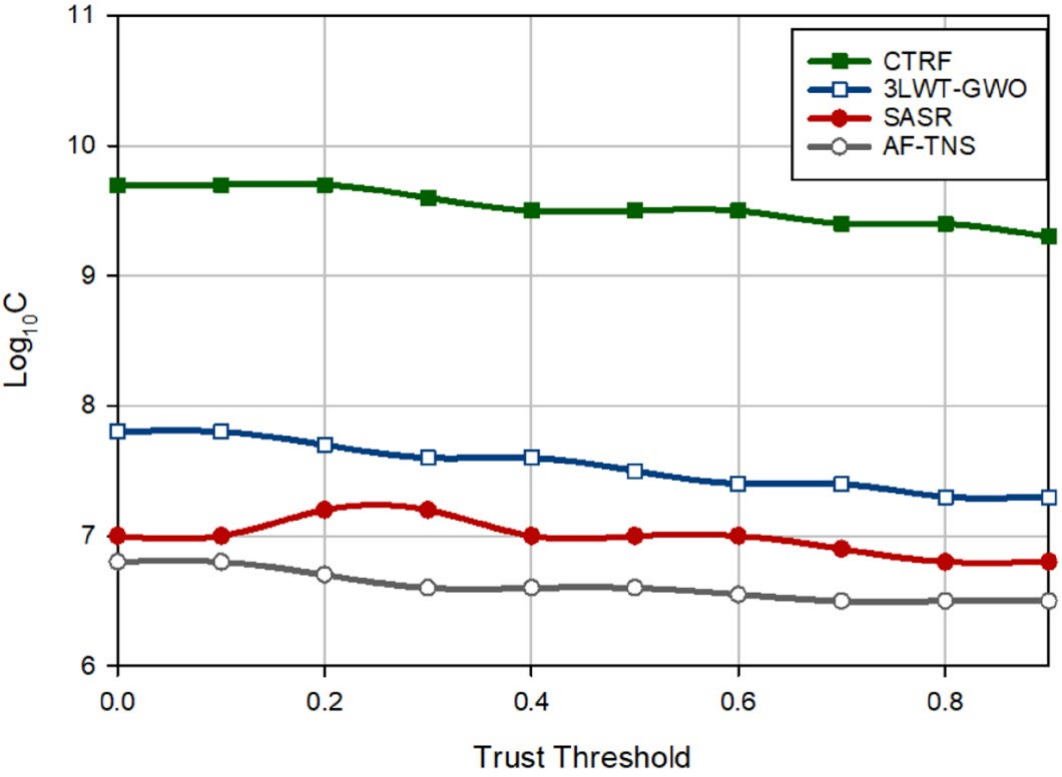
Comparison of communication costs in different approaches.

Figure 11 shows a comparison between communication costs in different schemes. This metric indicates the number of control messages sent by a node to deliver a packet to the target nod and evaluate the trust of nodes. CTRF reduces the communication cost by 26.39%, 36.36%, and 44.07% compared to 3LWT-GWO, SASR, and AF-TNS, respectively. This proves that CTRF has a very good performance in terms of overhead. This has a positive effect on the network lifetime. The main reason for this issue is that in the routing process, CTRF calculates a score based on the reliability of the route, the energy of the route, and the quality of the route for each route discovered between source-destination pairs and selects the route with the highest score to send data. These parameters help to choose stable paths in the network. As a result, the number of route failures is reduced and the need to rebuild failed routes is also reduced. This has a positive effect on reducing communication costs.

## Conclusion

This paper presented a clustered trust-aware routing protocol base on the fire hawk optimizer called CTRF. our approach contains three components: weighted trust mechanism (WTM), FHO-based clustering, and trusted routing. WTM estimates the trust of the nodes in accordance with weighted reception rate, weighted redundancy rate, and energy state. WTM utilizes a regulatory factor for each trust parameter to increase or decrease the trust level of nodes according to their hostile or friendly behaviors. In the clustering mechanism, the BS is responsible for choosing the best nodes among the high-energy and reliable nodes. In addition, this mechanism presents a new cost function based on intra-cluster and inter-cluster distances, the distance from CHs to BS, residual energy, and the size of clusters. Finally, the routing process has responsibility to select high quality, reliable, high-energy paths. The evaluations made in this paper show that CTRF has the best residual energy, throughput, packet loss rate, delay, detection rate, and accuracy. It improves the residual energy level by 6.44%, 9.79%, and 16.15%; throughput by 8.96%, 17.51%, and 43.09%; delay by 32.20%, 42.83%, and 58.7%; the detection rate by 2.07%, 3.58%, and 6.26%, and accuracy by 5.16%, 9.46%, and 14.64% compared to 3LWT-GWO, SASR, and AF-TNS, respectively. In future research directions, we attempt to improve the accuracy of the security mechanism designed in CTRF by adding new techniques such as neural networks (ANNs) and reinforcement learning (RF). Note that the WTM mechanism designed in CTRF is a powerful and accurate trust mechanism, but it does not have the ability of self-organization and self-adaptation. To solve this problem, ANNs and reinforcement learning techniques, especially Q-learning are useful solutions and can be used to design an adaptive trust mechanism in wireless sensor networks. In addition, the clustering algorithm will be designed using other optimization algorithms such as dragonfly algorithm (DA), gray wolf algorithm (GWO), and genetic algorithm (GA), and will be evaluated their effects on network performance.

## Data Availability

All data generated or analyzed during this study are included in this published article.
